# The tool effect is lower in older adults with or without cognitive impairments than in young adults

**DOI:** 10.1007/s00426-023-01872-2

**Published:** 2023-09-28

**Authors:** Marion Luyat, Kévin Dumez, Myriam Noël, Emin Altintas, Cédric Campion, Gilles Lafargue, Michel Guerraz

**Affiliations:** 1 Univ. Lille, ULR 4072 – PSITEC – Psychologie : Interactions, Temps, Emotions, Cognition, 59000 Lille, France; 2 Clinique du Val de Lys (Groupe Ramsay), 167 rue Nationale, 59200 Tourcoing, France; 3 Centre hospitalier de Lens, Unité de gériatrie, 99 route de la Bassée, 62300 Lens, France; 4 Univ. Reims, Laboratoire C2S EA 6291, Departement de Psychologie, 51000 Reims, France; 5 Univ. Grenoble Alpes, Univ. Savoie Mont Blanc, CNRS, LPNC, 38000 Grenoble, France; 6 Centre Hospitalier de Tourcoing, Unité de gériatrie, 59200 Tourcoing, France

## Abstract

Grabbing a phone from a table or stepping over an obstacle on the ground are daily activities that require the brain to take account of both object and the body’s parameters. Research has shown that a person’s estimated maximum reach is temporarily overestimated after using a tool, even when the tool is no longer in hand. This tool effect reflects the high plasticity of the perceptual-motor system (e.g., body schema updating)—at least in young individuals. The objective of the present study was to determine whether the tool effect is smaller in older adults. Forty-four young adults, 37 older adults without cognitive impairment and 30 older adults with cognitive impairment took part in the experiment. The task consisted in visually estimating the ability to reach (using the index finger) a target positioned at different locations on a table, both before and after using a rake. We observed a strong after-effect of tool use in the young adults only. Conversely, a tool effect was similarly absent in the older adults without and with cognitive impairment. Moreover, even before the tool was used, the maximum reach was overestimated in each of the three groups, although the overestimation was greatest in the two groups of older adults. In summary, we showed that the tool effect, observed in young adults, was absent in older adults; this finding suggests that with advancing age, the perceptual-motor system is less able to adapt to novel sensorimotor contexts. This lack of adaptation might explain (at least in part) the overestimation of motor skills often reported in the elderly.

## Introduction

Grabbing an object located a short distance from one’s chair and stepping over a small obstacle on the floor are daily actions that are rather easy for a young, healthy person but can become challenging for an older person; in some cases, these activities even become dangerous and might lead a very frail person to fall. In order to carry out these actions safely, accurate anticipation of the consequences of the planned action is essential. In the field of cognitive neuroscience, it has been suggested that the brain relies on internal models of planned actions (Jeannerod, 1994; Wolpert, 1997; Wolpert et al., 1995). More precisely, the forward model provides an estimate of possible outcomes of the upcoming action by taking account of anthropometric parameters of the body segments in a dynamic, whole-body configuration relative to the environment (i.e., the body schema).

Under normal circumstances, the brain continuously updates its internal models of action on the basis of the person’s experience and body parameters. This updating is particularly important during childhood when growth of the body requires the body schema to be rapidly adapted to new, greater dimensions (Barra et al., 2020; Cignetti et al., 2013). Like childhood, old age is a time of marked and varied changes in the body: loss of height, muscle mass and muscle strength (Stelmach & Hömberg, 1993). In order to interact optimally with the environment in older age, the brain’s internal models must take account of changes in body shape and posture and declines in sensorimotor and physical abilities. If the body schema is not updated properly, there will be a mismatch between what the person thinks she/he can do and what she/he can actually do (Lafargue et al., 2013).

How, then, can failure to update the predictive internal model in old age be investigated? In young adults, earlier research revealed a change in the maximum estimated reach after use of a rake to reach tokens on a table (Bourgeois et al., 2014). The main finding was that the participants verbally overestimated their maximum reach, even when they were no longer holding the rake. This showed that the use of the tool recalibrated the perceptual-motor system (i.e. the internal predictive model has been updated accordingly) and that this tool effect lasted for a time after use (i.e., the after-effect of tool use also called the “tool effect” below). Paradoxically, the “after-effect” of tool use observed in young people, which is “erroneous”, evidences in fact the high plasticity of the perceptual-motor system with regard to the new action capabilities offered by the tool. To our knowledge, such an experiment using a reaching task and focusing on the after-effect of tool use has not yet been conducted with older adults.

However, in young adults, holding a tool can specifically affect distance perception, even when only a distance estimation task is required, without any actual or imagined reaching task (Costello et al., 2015; Osiurak et al., 2012; Witt et al., 2005). As pointed by Carello et al. (1989; p. 29) (among the first researchers to test reachability from an ecological point of view and to comment on the computational/cognitivist approach): “*If perceiving what is reachable is a matter of computation, then the computation seems to divide into three steps: (a) computing the distance of the target object* (*e.g., by quantifying the* separation *between a point representing the person's position and a point representing the object's position*), *(b) computing the furthest possible extension of the limbs given the current posture and surface layout* (*e.g., by quantifying the separation between a point representing the proximal end of a body segment and a point representing the segment's distal end*), and *c) comparing the quantities yielded by the two computations*.” In this conventional computational model, the tool effect can be explained by two main factors: (i) elongation of the representation of the arm during or after tool use and/or (ii) a decrease in the perceived distance. Both factors could lead to overestimation of the subjective maximum reach.

For instance, Witt et al. (2005) studied distance perception in young individuals; the latter perceived targets to be closer when required to reach out with a stick than when required to reach out with their hands. The researchers concluded that “*targets within reach are perceived to be closer than targets beyond reach*” (Witt et al., 2005). Their explanation reconciles (in our opinion) the apparently opposing factors mentioned above, elongation of the representation of the arm and a decrease in the perceived distance. In Witt et al.’s proposal, reachability becomes the benchmark per se: holding a tool leads to a measurement of space in relation to action capabilities (i.e., reachability), and distances then appear to be shorter due to the extension of the effector by the tool what may also influence the perceived length of the arm itself. As stated by Gibson (1979/1986, p. 240): “*The continuous act of perceiving involves the coperceiving oneself*”. The fact that the person believes she/he can reach a longer distance than she/he actually can, even in the absence of the tool (although after having used it), probably reflects the time needed for the perceptual-motor system to update to the new context (e.g., the arm without the tool). Thus, metric space is not the standard used to scale both the body or the space around us; this standard is rather the person’s idiosyncratic capability to reach a certain distance (rightly called “reachability”), regardless of whether a tool is used or not. In this conception, body and space are measured relative to the action of reaching and so are action-scaled.

Using much the same method as Witt et al. (2005), Costello et al. (2015) reported that the effect of tool use on distance perception was smaller in older adults than in younger adults. It should be noted that in both studies, the tool is held in the hand during the task and consequently, after-effect of the tool use was not measured. Thus, combining the proposal of Witt et al. (2005) and the results of Costello et al. (2015), we would expect, in a reaching task, a decrease of the after-effect of the tool use in older adults, reflecting less plasticity of the perceptual-motor system to update the motor skills. When asked to estimate their subjective maximum reach with the index finger after tool use, older adults should be less sensitive to tool use (i.e., they should overestimate their maximum reach to a lesser extent) than younger adults. Measuring the after-effect of tool use is interesting because it reveals more about the updating process: as the tool is no longer in hand, the direct influence acting as a bias on the judgments cannot be evoked.

By the same logic, the plasticity of the perceptual-motor system may be further compromised in older adults with cognitive impairments. Although they differ in several qualitative aspects of cognitive function (for a review, see Toepper, 2017), age-related degenerative diseases such as Alzheimer’s disease can be considered as accelerated aging in several neurophysiological parameters (grey and white matter degeneration as well as to changes in neural activation, functional connectivity, and neurotransmission) (Dennis & Thompson, 2014). Moreover, older adults with cognitive impairment show worse motor imagery abilities (Bourrelier et al., 2015), motor dysfunction and present a higher risk of falls (Muir et al., 2012; Wu et al., 2016) relative to their peers without cognitive impairments. Several studies have also shown that motor dysfunction may precede the onset of dementia and its presence could predict adverse outcomes in patients with Alzheimer disease, such as fall risk (for a review, see Poirier et al., 2021). Thus, the objective of the present study was to confirm that the after effect of tool use was diminished in older adults in general and even more so in older adults suffering from cognitive impairment. In the task, the seated participants had to assess their maximum reachability limit before and after using a tool (a rake) to reach tokens on a table.

## Methods

### Participants

A total of 111 volunteers were included in the study. The study protocol was approved by the regional institutional review board (*CPP Nord-Ouest II*, Amiens, France) and registered in the European Union Drug Regulating Authorities Clinical Trials Database (reference: EudraCT 2014-A00129-38). All participants gave their prior written informed consent, and all the data were recorded anonymously. The group of healthy (control) older participants (OG_controls_) included 37 adults (32 women), the group of older participants with cognitive impairments (OG_patients_) included 30 adults (25 women) and the younger adult group (YG) included 44 adults (37 women) (Table 1 depicts the demographic characteristics of the participants). The OG_patients_ were recruited at two nursing homes associated with Roubaix General Hospital (Roubaix, France). The other participants were initially contacted through community groups: various clubs for the older adults, and university or hospital staff bodies for the younger adults. None of the participants suffered from major musculoskeletal or sensory problems. OG_controls_ and YG were autonomous in everyday life, including the ability to walk unaided.

**Table 1:** Demographic characteristics of the participants

Sample characteristics	*n*	Age (in years)				MMSE			
		*M*	SD	Min	Max	*M*	SD	Min	Max
Groups									
YG	44	29.98	9.56	18	53	30	0	30	30
OG_controls_	37	77.49	6.35	66	91	29.27	1.15	27	30
OG_patients_	30	80.43	8.12	65	96	21.77	2.65	17	26

*YG* the younger adult group, *OG*_*controls*_ the group of healthy (control) older participants, *OG*_*patients*_ the group of older participants with cognitive impairments, MMSE: Mini Mental State Examination

### Materials

The visual stimulus consisted of a black dot (diameter: 2.5 cm) projected on the surface of a rectangular table (183 × 122 cm, covered with a smooth white tablecloth) via an overhead projector attached 165 cm above the table top. The overhead projector was connected to a Fujitsu^®^ brand laptop computer. Given that several nursing homes had been invited to recruit participants, the experimental setup was portable. The visual stimulus was projected at 26 different locations relative to the edge of the table near the participant’s body (36, 38.9, 41.5, 44.5, 47, 49.8, 52, 55, 57.5, 60.5, 63.2, 65.5, 68.5, 71, 73.5, 76.5, 79, 81.5, 84, 86.5, 89.4, 92, 94.5, 97.9, 100, 102.5 cm) along the body midline for a duration of 500 ms and in random order, using Eprime^®^ software. For each participant, the system was calibrated to ensure that the projected stimulus corresponded to the correct projected distance. In order to avoid any visual and/or auditory distractors, no surrounding objects or stimuli were in the participant’s field of vision. Furthermore, the brightness of the room lighting was reduced during the projection so that the stimulus was as prominent and visible as possible.

A wooden rake (with a 36-cm-long handle) and plastic tokens (diameter: 2.5 cm, i.e., similar to that of the visual stimuli) were used in the tool-use phase. During this phase, the experimenter ensured that there was adequate lighting in the room because the projector no longer provided any light.

### Procedure

**Fig. 1:**
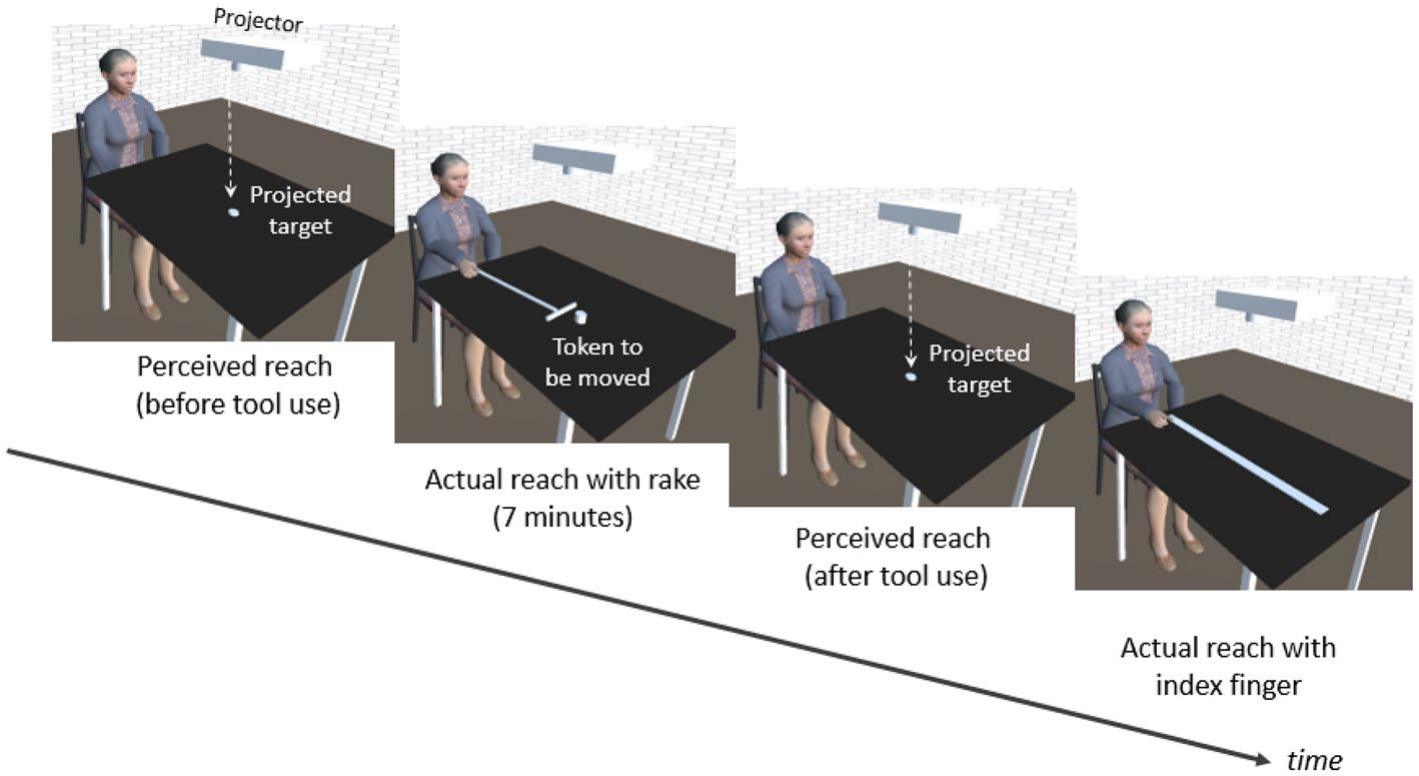
The sequential phases of the experimental procedure, with (from left to right) (i) a first verbal estimation of reachability (estimation 1), (ii) tool use, in which the rake was used to reach tokens, (iii) a second verbal estimation of reachability without the rake in hand (estimation 2), and (iv) measurement of the actual maximum reach with the index finger

The procedure was adapted from the study by Carello et al. (1989). Each participant was tested individually in a quiet room. The experiment consisted of four sequential phases; depicted in Fig. 1: (i) a first verbal estimation (“yes” or “no” responses) of reachability before tool use (estimation 1), (ii) the 7-min tool-use phase, which consisted in reaching out for tokens on the table with the rake and dragging them back towards the body, (iii) a second verbal estimation (“yes” or “no” responses) of reachability without the rake in hand (estimation 2), and (iv) measurement of the actual maximum reach with the index. For the two verbal pre- and post-estimations, we determined the perceptual threshold using a logistic regression model that best fitted the reachable/unreachable responses. This threshold is the boundary between nonreachable distances and reachable distances and corresponds to be the maximum distance that the participant estimates that she/he can reach with the index finger.

The actual maximum reach with the index finger was measured after the verbal estimates, in order to prevent immediate improvement of the subsequent estimation by practice (as evidenced by Yasuda et al. (2014)). In estimations 1 and 2 (i.e., before and after tool use), the 26 different locations of the projected stimulus onto the table were presented in a random order. However, this order was the same for all participants. In order to avoid tiring the particularly frail and easily fatigued participants with cognitive impairment, and to maximize the probability of observing an after-effect (the tool effect) of rake use, the 26 different locations were presented only once. Throughout the experiment, the participants did not receive any feedback on the accuracy of their judgments. It should be noted that participants were seated with their back against the chair and were required to stay sitting in that way for the duration of the experiment. During verbal estimates, the participants were instructed to use mental imagery of their arm movement only and with their back against the seat; this was to avoid judgments based on whole-body engagement and thus overestimation of the prehensile space (see Rochat & Wraga, 1997).

### Data processing and statistical analysis

In order to measure misjudgments of maximum reachability, we calculated two overestimation indexes for each participant by subtracting the actual maximum reach (obtained in the fourth phase) from the estimated maximum reach before tool use (estimation 1) or from the estimated maximum reach after tool use (estimation 2). The statistical analysis of the overestimation indexes was based on an analysis of variance (ANOVA) with *group* (YG, OG_controls_ and OG_patients_) as a between-category factor and *estimation* (estimation 1 and estimation 2) as a repeated measure. The Newman–Keuls method was used for post hoc tests. The threshold for statistical significance was set to *p* = 0.05. Size effects were reported with partial eta squared statistics ($\eta_{{\text{p}}}^{2}$).

## Results

**Fig. 2:**
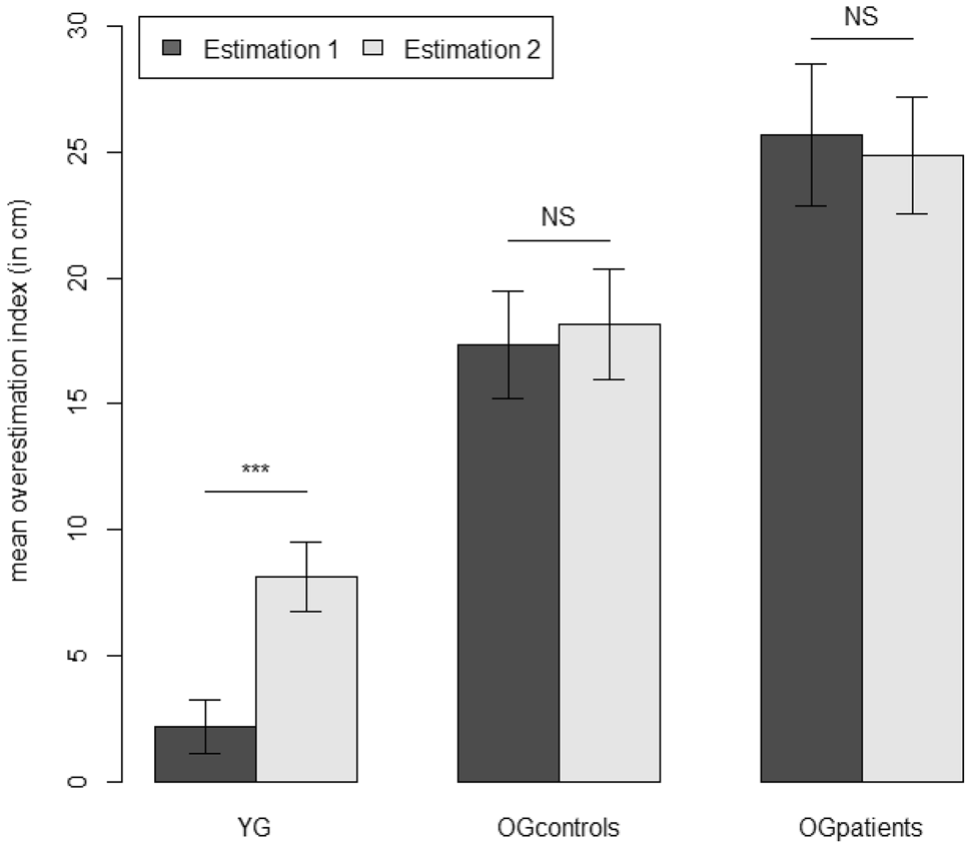
Means of overestimation index (estimated maximum reach minus actual maximum reach, in cm) as a function of estimation (estimation 1: before tool use, estimation 2: after tool use) and the participant group (*YG* younger adults, *OG*_*controls*_ older adults without cognitive impairment, *OG*_*patients*_ older adults with cognitive impairment). The error bars correspond to the SEM

The means of overestimation index and standard errors of the mean (SEM) for each study group in each estimation (1 or 2) are shown in Fig. 2. The ANOVA revealed a significant effect of *group*: *F*_(2, 108)_ = 28.88; *p* < 0.001; $\eta_{{\text{p}}}^{2}$ = 0.35). As shown in Fig. 2, the older participants with cognitive impairments overestimated their maximum reach more (OG_patients:_: *M* = 25.28 cm; SEM = 2.11 cm) than the older control participants did (OG_controls_: *M* = 17.76 cm; SEM = 1.90 cm), who in turn overestimated their maximum reach more than the younger participants did (YG: *M* = 5.16 cm; SEM = 1.74). Newman–Keuls post hoc tests confirmed that the pairwise differences in means were consistently significant (*p* values < 0.01). The ANOVA also revealed a significant effect of *estimation*, *F*_(1,108)_ = 9.52; *p* < 0.003; $\eta_{{\text{p}}}^{2}$ = 0.08, with an estimated maximum reach greater after tool use (*M* = 17.06 cm; SEM = 1.17 cm) than before tool use (*M* = 15.04 cm; SEM = 1.14 cm). Lastly, the interaction between *group* and *estimation* was significant: *F*_(2,108)_ = 10.52; *p* < 0.001; $\eta_{{\text{p}}}^{2}$ = 0.16. Indeed, a pre-planned contrast analysis showed that the difference between the estimated maximum reach after tool use (estimation 2) and before tool use (estimation 1) (i.e., the tool effect; see Fig. 2) was significant for the YG (*F*_(1,108)_ = 34.44; *p* < 0.001) but not for the two groups of older participants: OG_controls_: *F*_(1,108)_ = 0.53; *p* = 0.47; OG_patients_: *F*_(1,108)_ = 0.40; *p* = 0.53. In other words, these results indicate that a tool effect was present in the YG but not in the OG_controls_ or OG_patients_.

**Fig. 3:**
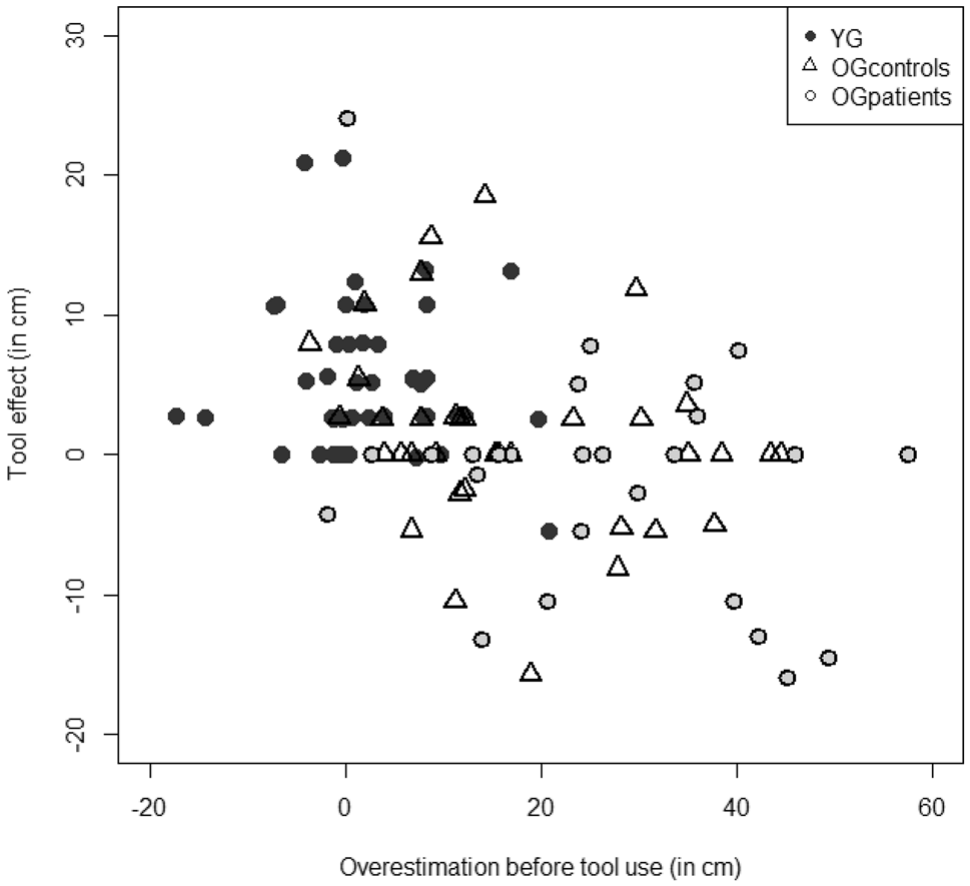
Scatter plot representing the relationship between the overestimation of the maximal reach before tool use in cm and the tool effect in cm in young participants (YG), healthy older participants (OG_controls_) and older participants with cognitive impairments (OG_patients_)

A correlation analysis of pooled data from all the participants highlighted a negative correlation between the overestimation indexes measured before tool use and the tool effect (i.e., the difference between the estimates before and after tool use): *r* = − 0.42; *n* = 111; *p* < 0.001). However, when the different groups were considered separately, the correlation was significant only for OG_patients_ (OG_patients_: *r* = − 0.36; *p* = 0.05; OG_controls_: *r* = − 0.22; *p* = 0.19; YG: *r* = − 0.02; *p* = 0.91), i.e., the group with both the greatest overestimation index before tool use and the lack of a tool effect. A scatter plot for all the pooled participants is shown in Fig. 3.

## Discussion

The primary objective of the present research was to establish whether the tool effect (i.e., a greater verbal estimate of subjective maximum reach with the index finger after tool use) was lower in older adults (and especially those with cognitive impairments) than in younger adults. The task used in the present study consisted of a verbal assessment of the subjective maximum reach without performing the real action both before (estimation 1) and after (estimation 2) the participants had used a rake to reach tokens. It is important to note that in both estimations, the participant was not holding the tool at the time when she/he was asked to verbally state her/his maximum reach. Thus, what was studied here, in priority, was the after-effect of tool use.

First, our results showed a clear, statistically significant tool effect in the younger participants, i.e., a greater estimated maximum reach after tool use than before—although the tool was no longer being held. The tool effect in the YG was globally of the same magnitude as that reported by Bourgeois et al. (2014). More interestingly, and in support of our main hypothesis, we did not observe a tool effect in the two groups of older adults. This lack of a tool effect suggests that the plasticity of the perceptual-motor system may be compromised by advancing age. As mentioned in the introduction, Costello et al. (2015) reported that the effect of tool use on distance perception was smaller in older adults than in younger adults; in other words, the older adults were less sensitive to tool use in the distance perception task. However, despite several important differences compared to how we proceeded, this result fits well with the smaller tool effect among older adults observed in the present study.

One might expect the decline in perceptual-motor adaptation to be more pronounced in OG_patients_ than in healthy older participants. However, the absence of a tool effect in the OG_controls_ in the present experiment, possibly reflecting a floor effect, may have precluded the observation of an additional effect related to cognitive impairment. The absence of a significant after-effect of the tool use in the older groups could reflect changes to the aging brain (see Costello et al., 2015). Memory loss, even at a procedural level, is also typical of elderly and could partly explain the absence of after-effect of tool use. Bernard and Seidler (2014) proposed that internal models of action are affected by age, especially forward modeling. These authors proposed that age-related defect in the formation of new internal models and/or the degradation of existing models could be explained by age-related changes in cerebellar functioning and/or a disruption of its connections to cortical motor areas, and the basal ganglia (for a review, see Kuehn et al., 2018). Moreover, although the existing literature mainly focuses on the pathology of the medial temporal lobe, neuroimaging studies have shown now a clear involvement of the parietal cortex in mild cognitive impairments and Alzheimer disease (for a review, see Jacobs et al., 2012). Parietal-hippocampal network could be involved in the tool after-effect decrease in older participants since parietal-hippocampal rTMS appears to improve cognitive function, in particular memory, in Alzheimer’s disease (Wei et al., 2022).

However, the lack of an after-effect of tool use in the older groups could also be due to methodological factors such as insufficient testing in estimations 1 and 2. The tool-use phase may also prevent the after-effect from occurring if insufficient. However, this phase lasted seven minutes, which is rather long for the participant, a maximum in fact, especially for the older ones, because it is a monotonous task that, in addition, involves the shoulder joint. The procedure should be improved in the future, for example by using other psychophysical methods that require fewer trials. The number and variety of tasks during the tool-use phase could also be increased, which would reduce monotony and perhaps promote the emergence of a tool effect, if it exists in older adults.

Another interesting result raised by the present study was the overestimation of maximum reach found before tool use in estimation 1. This overestimation was found in all three groups but was pronounced in OG_controls_ control group and even more so in OG_patients_. Older adults often overestimate their performance in motor imagery tasks (Caffier et al., 2019; Gabbard et al., 2011; Lafargue et al., 2013; Liu-Ambrose et al., 2008; Okimoto et al., 2017; Robinovitch & Cronin, 1999; Sakurai et al., 2013, 2016, 2017). Regarding the reachability task more specifically, our results are well in line with Gabbard et al.’s (2011) report whereby seated older adults overestimated their maximum reachability more than younger adults did. The question of which processes underlie this overestimation then arises. As noted above, brain changes in normal and majored pathological aging could explain the fact that OG_patients_ showed more pronounced overestimation by over optimistic predictions about future actions.

Interestingly, our results showed that overestimation was negatively correlated with the magnitude of the tool effect: the higher the overestimation, the lower the tool effect is. However, the analysis per group showed that this correlation was significant only in OG_patients_. As suggested by some researchers but not yet demonstrated (Gabbard et al., 2011; Lafargue et al., 2013), overestimation might be responsible for loss of balance or falls, which are known to increase with age and especially so in older adults with cognitive impairments (Muir et al., 2012; Wu et al., 2016). Ideally, internal models must take account of age-related declines in sensorimotor and physical abilities. Failure by older adults to update their internal models could lead to overestimation of motor and postural capabilities and thus to overly optimistic predictions about upcoming behaviors. For example, older adults might attempt to walk on surfaces that appear to them to be safe but on which they would be unable (given their reduced motor skills) to stand. Even in a seated position, a poor assessment of reachability when trying to reach for a telephone or an object on a table (for instance) could lead to imbalance and even falling out of the chair. Hence, overestimation of postural capabilities can be a major risk factor in falls in the elderly. In this respect, future research could usefully combine a reaching task with a distance perception task and a questionnaire on the fall risk. Adding a balance test or postural sway measurements would also be very informative.

In summary, our present results showed that the tool effect observed in younger adults is absent in older adults. This finding is in line with the assumed age-related decline in the perceptual-motor system’s ability to adapt to novel sensory motor contexts. In turn, this decline might explain the misjudgments of motor skills often reported in the elderly.

## Data Availability

Data have been made available from the following address https://figshare.com/s/47eb394ad05e1b550dbc with 10.6084/m9.figshare.21554625.
